# Systematic review and meta-analysis of the diagnostic accuracy of ultrasonography for deep vein thrombosis

**DOI:** 10.1186/1471-2342-5-6

**Published:** 2005-10-03

**Authors:** Steve Goodacre, Fiona Sampson, Steve Thomas, Edwin van Beek, Alex Sutton

**Affiliations:** 1 School of Health, University of Sheffield, Regent Court, 30 Regent Street, Sheffield, S1 4DA, UK; 2 Academic Vascular Unit, University of Sheffield, Coleridge House, Northern General Hospital, Herries Road, Sheffield, S5 7AU, UK; 3 Carver College of Medicine, University of Iowa Hospitals and Clinics, Department of Radiology, 3895 JPP, 200 Hawkins Drive, Iowa City, IA 52242-1077, USA; 4 Department of Health Sciences, University of Leicester, 22-28 Princess Road West, Leicester, LE1 6TP, UK

## Abstract

**Background:**

Ultrasound (US) has largely replaced contrast venography as the definitive diagnostic test for deep vein thrombosis (DVT). We aimed to derive a definitive estimate of the diagnostic accuracy of US for clinically suspected DVT and identify study-level factors that might predict accuracy.

**Methods:**

We undertook a systematic review, meta-analysis and meta-regression of diagnostic cohort studies that compared US to contrast venography in patients with suspected DVT. We searched Medline, EMBASE, CINAHL, Web of Science, Cochrane Database of Systematic Reviews, Cochrane Controlled Trials Register, Database of Reviews of Effectiveness, the ACP Journal Club, and citation lists (1966 to April 2004). Random effects meta-analysis was used to derive pooled estimates of sensitivity and specificity. Random effects meta-regression was used to identify study-level covariates that predicted diagnostic performance.

**Results:**

We identified 100 cohorts comparing US to venography in patients with suspected DVT. Overall sensitivity for proximal DVT (95% confidence interval) was 94.2% (93.2 to 95.0), for distal DVT was 63.5% (59.8 to 67.0), and specificity was 93.8% (93.1 to 94.4). Duplex US had pooled sensitivity of 96.5% (95.1 to 97.6) for proximal DVT, 71.2% (64.6 to 77.2) for distal DVT and specificity of 94.0% (92.8 to 95.1). Triplex US had pooled sensitivity of 96.4% (94.4 to 97.1%) for proximal DVT, 75.2% (67.7 to 81.6) for distal DVT and specificity of 94.3% (92.5 to 95.8). Compression US alone had pooled sensitivity of 93.8 % (92.0 to 95.3%) for proximal DVT, 56.8% (49.0 to 66.4) for distal DVT and specificity of 97.8% (97.0 to 98.4). Sensitivity was higher in more recently published studies and in cohorts with higher prevalence of DVT and more proximal DVT, and was lower in cohorts that reported interpretation by a radiologist. Specificity was higher in cohorts that excluded patients with previous DVT. No studies were identified that compared repeat US to venography in all patients. Repeat US appears to have a positive yield of 1.3%, with 89% of these being confirmed by venography.

**Conclusion:**

Combined colour-doppler US techniques have optimal sensitivity, while compression US has optimal specificity for DVT. However, all estimates are subject to substantial unexplained heterogeneity. The role of repeat scanning is very uncertain and based upon limited data.

## Background

Deep vein thrombosis (DVT) is an important cause of mortality and morbidity that requires accurate diagnosis. Ultrasound (US) examination has now largely replaced contrast venography as the standard test for diagnosing clinically suspected DVT [1]. Numerous studies have compared US to contrast venography in patients with clinically suspected DVT. These were most recently summarised by Kearon in 1998 who concluded that US had a sensitivity of 97% for proximal DVT, 72% for distal DVT and a specificity of 94% [2].

Meta-analytic techniques have developed rapidly in recent years. There is increasing recognition that the results of individual studies of a diagnostic test are often subject to substantial heterogeneity and that methodological factors may influence the results of studies [3,4]. Statistical techniques, such as meta-regression, allow researchers to explore data from systematic reviews for evidence that study-level covariates may influence diagnostic accuracy. There is also an increasing recognition that systematic reviews of diagnostic test data may be subject to publication bias, [4] although solutions to this problem, such as registries of studies, have yet to be developed.

Since US is now established as a definitive diagnostic test for DVT it is unlikely that many new studies evaluating the diagnostic accuracy of US will be forthcoming. This therefore represents an opportune time to undertake a definitive systematic review, meta-analysis and meta-regression of the diagnostic accuracy of US for clinically suspected DVT. We aimed to estimate the sensitivity and specificity of US for DVT, identify study-level covariates that are associated with variation in sensitivity and specificity, and seek evidence of publication bias in diagnostic studies of US for DVT.

## Methods

We sought to identify all diagnostic cohort studies of patients with clinically suspected DVT who underwent testing with US followed by a reference standard of contrast venography. We searched Medline, EMBASE, CINAHL, Web of Science, Cochrane Database of Systematic Reviews, Cochrane Controlled Trials Register, Database of Reviews of Effectiveness, and ACP Journal Club (1966 to April 2004). The bibliographies of all articles selected for the review were scanned for potentially relevant articles that were not identified by the original search.

Two reviewers (FS and SG) screened the titles and abstracts of all articles to independently identify potentially relevant articles. Full copies of all selected articles were retrieved and reviewed by the same two reviewers, who independently selected relevant articles. At both stages of selection a Kappa score was calculated and disagreements resolved by discussion. Studies published in English, French, Spanish, Italian or German were included. Studies published in other languages were excluded. Abstracts and letters were included if they reported data in sufficient detail to allow inclusion in the analysis. If not, the authors were contacted and asked to provide details of the data or any full publications.

We specifically excluded case-control studies, in which US results in a group of patients with DVT were compared to a control group of patients without DVT; studies that used a reference standard other than venography; studies with less than ten patients; and studies of patients with suspected pulmonary embolus. Although we collected data from cohorts of asymptomatic patients and mixed cohorts (symptomatic and asymptomatic) we have only reported data here from patients with clinically suspected DVT. The role of US in asymptomatic patients has recently been systematically reviewed [5].

Two independent reviewers (ST and EvB) extracted the following data from the selected studies onto a standardised proforma: the setting for patient recruitment, any exclusion criteria, population demographics, whether recruitment was consecutive and/or data collection prospective, which US technique was used, the US operator, and the number of true positives (proximal and distal), true negatives, false positives and false negatives (proximal and distal), either as reported or calculated from the reported data. The same two reviewers also independently determined whether US was interpreted by observers blind to the venogram result, and whether venography was interpreted by observers blind to the results of US. Discrepancies were checked and resolved by an independent reviewer (FS). If it was not possible to extract the necessary data from the published report we contacted the authors for clarification. We reviewed the data reported by each study and removed studies that contained duplicated data.

## Statistical analysis

Random effects models were used to estimate overall sensitivity and specificity, and a Chi-square test for heterogeneity between studies. Where 0 counts occurred for study data, a continuity correction of 0.5 was added to every value for that study in order to make the calculation of sensitivity and specificity defined. These analyses were undertaken using MetaDiSc statistical software [6] and further details of the models fitted is given elsewhere [7]. Initially all studies were analysed together and random effects meta-regression undertaken to identify potential causes of heterogeneity for sensitivity and specificity separately [8] (analysis carried out in STATA). Any covariate that showed an association with sensitivity or specificity (p < 0.1) was selected, and subgroups of studies identified by such covariates were meta-analysed separately. We decided, a priori, to undertake separate analyses of different US techniques: 1) Compression US only; 2) Colour Doppler only; 3) Continuous wave Doppler only; 4) Duplex (combined compression and colour Doppler US); 5) Triplex (combined compression, colour Doppler and continuous wave Doppler US).

Funnel plots were used to explore for evidence of publication bias. For both sensitivity and specificity the standard error of the log odds of the parameter was plotted against the log odds [9].

### Repeat or serial US

Repeat or serial US is often used to identify distal DVT, missed by the initial scan, that extend proximally and may thus be detected by US after an appropriate time delay (usually one week). We sought to identify studies of repeat or serial US in the main systematic review. However, we realised that we were unlikely to identify many studies that fulfilled our inclusion criteria, because of the logistic and ethical difficulties of asking patient to undergo successive US examinations followed by contrast venography. We therefore recorded separately any studies that reported use of serial or repeat US with clinical follow-up of patients, but which did not perform venography in all (or any) patients. Analysis simply consisted of recording the number of positive initial and repeat scans to estimate the yield of positive repeat scans.

## Results

**Figure 1:**
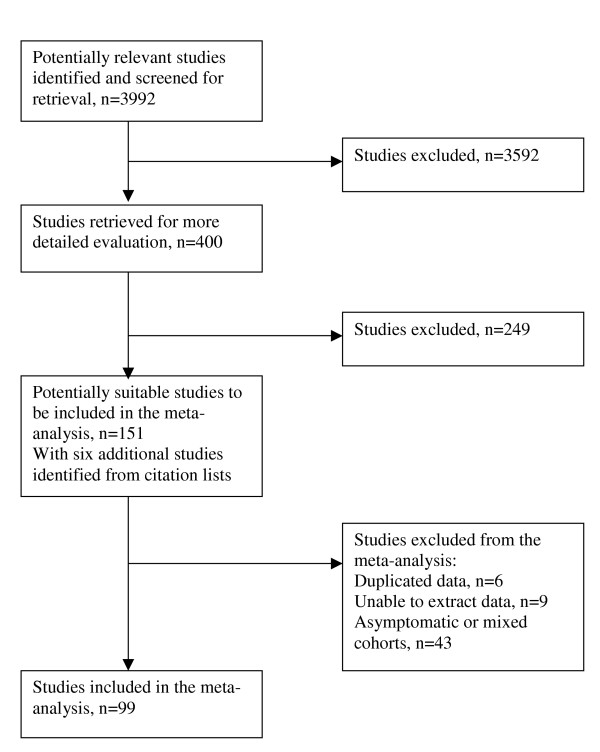
Flow diagram of studies considered for the review.

The flow of articles is outlined in figure 1. We scanned 3992 titles/abstracts and selected 400 potentially relevant articles for retrieval (kappa = 0.85). Review of the full articles identified 151 that met the inclusion criteria (kappa = 0.90). Review of the bibliographies of the selected articles identified six additional articles for inclusion. Six articles duplicated data published elsewhere and were excluded. We were unable to extract or analyse appropriate data from a further nine articles, despite attempts to contact the authors. Some 43 articles reported asymptomatic or mixed cohorts, so 99 articles were included in the meta-analysis. One article reported two cohorts, so the meta-analysis included a total of 100 cohorts [10-108].

### Characteristics of the included cohorts

The studies reported a total of 10323 patients, with cohorts varying in size from 11 to 847 patients (median N = 72). The studies varied in the way they reported their findings: 53 reported proximal and distal DVT separately, 19 only reported proximal DVT, three only reported distal DVT, and 25 were unclear or reported proximal and distal DVT together. DVT prevalence varied from 20% to 94% (median 48%). The proportion of proximal DVT (of all DVT detected) ranged from 48% to 100% (median 78%). The mean or median age was reported by 60 studies, and ranged from 39 to 68 (median 57). The male to female ratio was reported by 65 studies, with the proportion of males ranging from 15% to 95% (median 45%).

Cohorts were recruited from the following settings: outpatient clinic-11, inpatients-12, emergency department-4, mixed-18, and not stated-55. Recruitment was reported to be consecutive in 48, and prospective in 67. Twelve cohorts excluded patients with previous DVT, while 45 papers did not report any exclusion criteria. The following techniques were used: 22 used compression ultrasonography alone, five used Colour Doppler alone, 16 used continuous wave Doppler alone, 25 used triplex, 28 used duplex, and four used other techniques. Ultrasound was interpreted blind to the results of venography in 62 cohorts and was unclear in 38. Venography was interpreted blind to the ultrasound result in 56 cohorts, was interpreted by observers aware of ultrasound result in two, and was unclear in 42.

### Results of meta-analysis

**Figure 2:**
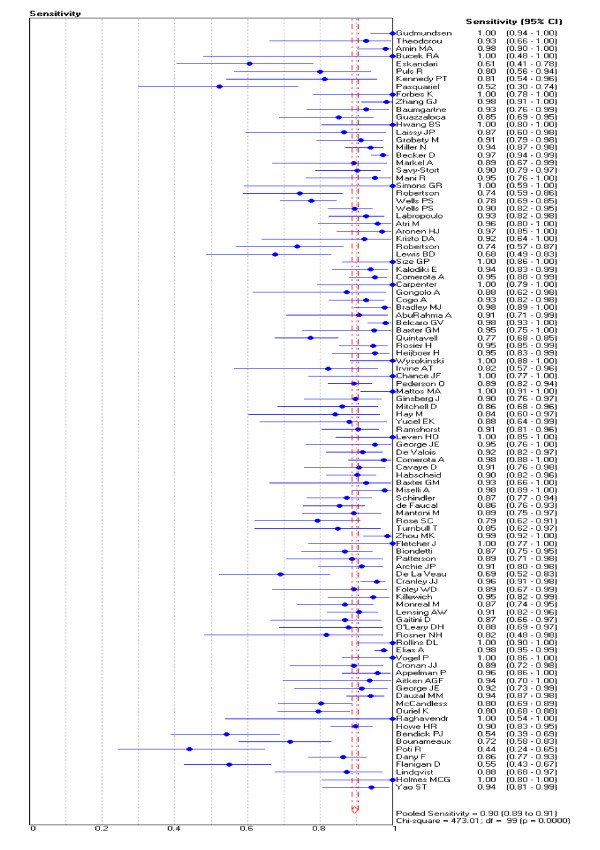
Forest plot of sensitivity.

**Figure 3:**
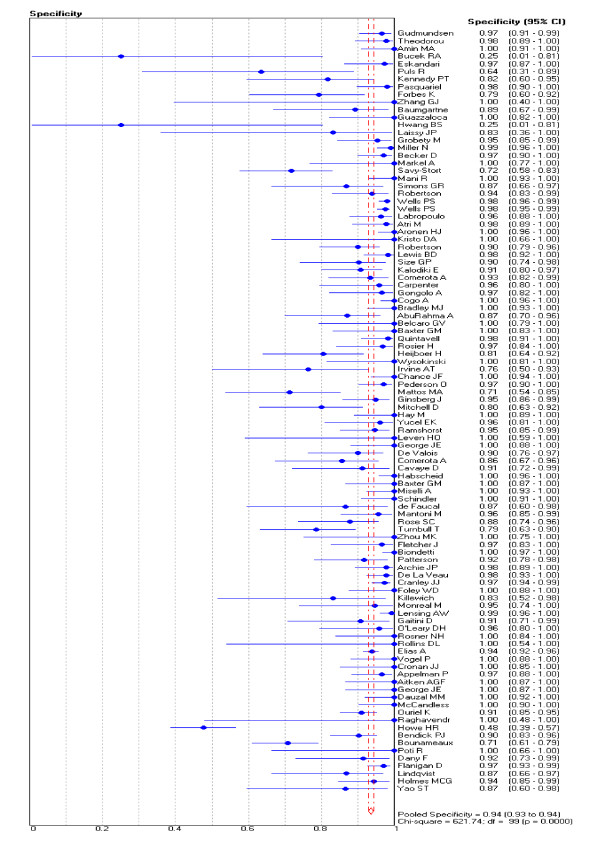
Forest plot of specificity.

Figures 2 and 3 show the Forest plots of sensitivity and specificity respectively. Point estimates of sensitivity and specificity are plotted, with 95% confidence intervals, for each cohort. Pooled sensitivity (95% CI, p-value for heterogeneity) for detecting any DVT was 89.7% (88.8 to 90.5, p < 0.001). Pooled sensitivity for detecting proximal DVT was 94.2% (93.2 to 95.0, p < 0.001) and for distal DVT was 63.5% (59.8 to 67.0, p < 0.001). Pooled specificity, calculated using data from all the studies, was 93.8% (93.1 to 94.4, p < 0.001). When restricted to the 53 studies reporting full data specificity was 94.2% (93.4 to 95.0, p < 0.001). Great care should be taken when interpreting these estimates because of the substantial heterogeneity. It may be argued that calculating summary estimates in these circumstances is inappropriate. However, it does provide a useful baseline from which to explore heterogeneity.

### Results of meta-regression

We undertook random effects meta-regression to identify possible causes for the heterogeneity. The results of meta-regression are outlined in Table 1. Using a threshold of p < 0.1 for statistical significance, interpretation by a radiologist, prevalence of DVT, the proportion of proximal DVT and date of publication were all significant predictors of sensitivity. The only significant predictor of specificity was exclusion of patients with a previous history of DVT.

More recently published studies, those with a higher prevalence of DVT and those with a higher proportion of proximal DVT tended to have higher sensitivity. There were 33 studies in which the operator was reported as being a radiologist. Meta-analysis showed that that diagnostic performance was generally slightly worse among these studies. Overall sensitivity (95% CI) was 86.1% (83.8 to 88.3), sensitivity for proximal DVT was 94.4% (92.3 to 96.1), sensitivity for distal DVT was 62.6% (55.4 to 69.4), and specificity was 92.4% (90.9 to 93.7). Twelve cohorts reported excluding patients with previous DVT. Meta-analysis showed that that specificity was higher amongst these cohorts: 97.6% (96.6 to 98.3).

Table 2 shows pooled estimates of sensitivity and specificity stratified by US technique used. Optimal sensitivity is achieved by using duplex or triplex, while optimal specificity is achieved by using compression alone.

### Funnel plots

**Figure 4:**
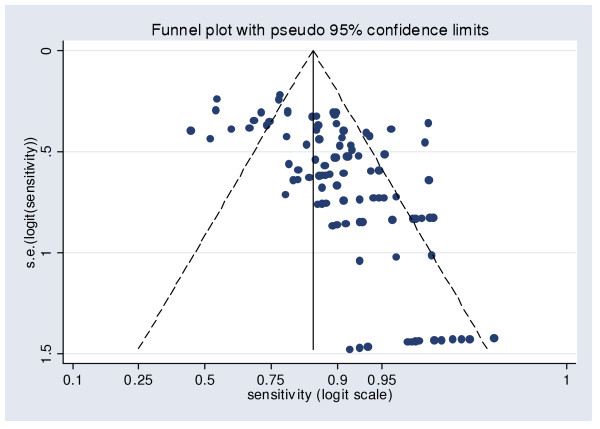
Funnel plot for sensitivity.

**Figure 5:**
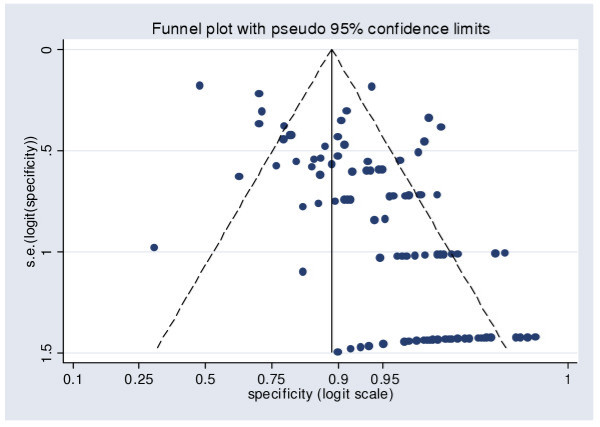
Funnel plot for specificity.

These are shown in figure 4 (sensitivity) and figure 5 (specificity). Both plots are asymmetrical, suggesting that smaller studies tend to report higher sensitivity and specificity. One possible explanation of this is publication bias. Smaller studies reporting lower sensitivity or specificity may be less likely to be submitted or accepted for publication.

### Repeat or serial US

We did not identify any studies that compared the results of repeat or serial scanning to venography in a complete cohort of patients, so none were included in the meta-analysis. However, we did identify several studies that reported the results of repeat US: five studies used repeat scanning for unselected cohorts with suspected DVT, [109-113] while four used repeat scanning for selected groups, based on the results of clinical risk scoring or D-dimer measurement [114-117] Three studies used venography in some patients to confirm the results of positive repeat scanning [109,112,113]. Results from these studies are summarised in Table 3.

In unselected cohorts repeat scanning had a positive yield of zero to 2%. Where venography was used to confirm positive findings, the positive predictive value of ultrasound was 82 to 94%. Overall, our best estimate of the positive yield of repeat scanning in unselected patients is 35/2610 (1.34%; 95% CI 0.97 to 1.86%) with a positive predictive value of 146/164 (89.0%; 95% CI 83.3 to 92.9%).

When repeat scanning is restricted on the basis of clinical probability or D-dimer the results suggest a higher yield of positive scans, although none of the studies used venographic confirmation. Two studies of repeat ultrasound limited to patients with a positive D-dimer produced an overall positive scan yield of 22/606 (3.63%; 95% CI 2.42 to 5.44%) [116,117].

## Discussion

The diagnostic accuracy of US for DVT varies according to the technique used. Optimal sensitivity is achieved by using duplex (proximal sensitivity 96%, distal sensitivity 71%, specificity 94%) or triplex US (proximal sensitivity 96%, distal sensitivity 75%, specificity 94%). Optimal specificity is achieved by using compression US alone (proximal sensitivity 94%, distal sensitivity 57%, specificity 98%). These findings suggest that compression US alone is probably the appropriate technique for most patients, if scanning is aimed simply at identifying proximal DVT. Most patients have a low probability of DVT, so optimal specificity is required to avoid generating excessive false positive results. However, when evaluating patients at high risk of DVT, or if scanning aims to identify distal DVT, then duplex or triplex US will probably be the appropriate technique.

Beyond US technique we identified few study-level predictors of sensitivity or specificity. Sensitivity tended to be higher in more recent studies, probably reflecting developing technology and expertise. Sensitivity was surprisingly lower in studies where scans were interpreted by a radiologist. This may be because these studies were more likely to use techniques at an earlier stage in their development. Another cause could be that compression ultrasonography is the simplest technique, whereas Doppler and colour US techniques are more challenging and therefore more likely result in greater reporting variability. The association between proportion of proximal DVT and sensitivity is unsurprising as US has better sensitivity for proximal DVT. The association between DVT prevalence in the study cohort and sensitivity may be explained by a similar mechanism. Selection of a cohort with a higher prevalence of DVT is likely to involve selection of cases with more easily detectable (i.e. larger and more proximal) DVT. Prevalence has been shown to be associated with variation in the performance of other diagnostic tests for DVT. Heim et al [118] showed that D-dimer has poorer accuracy in cohorts with a higher prevalence of DVT, probably due to lower specificity.

We identified no studies to reliably estimate the diagnostic accuracy of repeat scanning in comparison to contrast venography. Our best estimate of the diagnostic value of repeat scanning is that, in unselected patients with suspected DVT, it will have a positive yield of 1.3%, of whom 89% will be true positive and 11% false positive. A higher yield may be achieved by limiting repeat scanning to patients with a high clinical risk score and/or positive D-dimer. Whether these yields of positive scanning justify use of repeat scanning depend upon our estimates of the costs, benefits and risks of treating, or not treating, cases of DVT.

This study has some limitations that need to be considered. We did not search for unpublished data or studies published in languages other than English, French, Spanish, Italian or German. Studies of diagnostic tests are relatively easy to undertake, are often unfunded, and are not usually recorded on research registries. It is therefore unsurprising that systematic reviews of diagnostic test data rarely search for unpublished data [4] and that the potential effect of publication bias is unknown. Funnel plots for sensitivity and specificity were both asymmetrical. One possible explanation for this is that small studies reporting poor sensitivity or specificity may be less likely to be submitted or accepted for publication. If this is the case then the values for pooled sensitivity and specificity may represent over-estimates.

Despite undertaking meta-regression and stratifying results by US technique our findings were subject to significant unexplained heterogeneity. This heterogeneity is probably due to factors that were inadequately reported in the primary studies and therefore could not be explored in meta-regression. These factors include the characteristics of patients recruited (such as the prevalence of previous thromboembolism, obesity and co-morbidities), the training and experience of US operators, specific features of the US technique (such as the US frequency used), and any time delay between scanning and venography. These factors may have had a substantial influence upon sensitivity and specificity that will not have been identified in our analysis. Poor reporting also limited our ability to explore the effect of study design upon results. Use of blinding was often not described, studies rarely reported how uncertain or equivocal test results were handled, and the median prevalence of DVT in the cohorts (48%) suggests selective sampling of patients. These methodological weaknesses in the primary studies constitute a weakness in our meta-analysis.

The findings relating to repeat US scanning are subject to even greater limitations. Only a relatively small number of studies were identified and none compared repeat US to a venography in all cases. The potential benefit of repeat US is therefore very uncertain.

A potential clue to the influence of patient characteristics upon sensitivity and specificity is provided in a study by Wells et al [119], who reported their results stratified by the patient's clinical risk score into high, intermediate or low risk. Among patients with a high Wells score sensitivity (95% CI) was 91% (81 to 96) and specificity was 100 % (77 to 100). Among patients with an intermediate Wells score sensitivity was 61% (46 to 74) and specificity was 99% (94 to 100). Among patients with a low Wells score sensitivity was 67% (42 to 85) and specificity was 98% (95 to 99). This suggests that US sensitivity may be dependent upon clinical probability of DVT and concurs with our finding that sensitivity was higher in cohorts with higher prevalence.

The widespread current use of US to diagnose DVT is not based upon diagnostic cohort studies alone, but also upon management studies, in which cohorts of patients with negative US results are not treated, but followed up to identify evidence of missed thromboembolism. Studies of serial US [109-112,120], a single full-leg US [121-124], or US as part of a diagnostic algorithm [114,116,117,125-129] have shown low rates of thromboembolism during three to six month follow up. This suggests that, although our meta-analysis has shown that US does not have perfect sensitivity for DVT (especially distal thrombus), this does not translate into high rates of adverse outcome. This may be because application of a reasonably sensitive test to a population with low disease prevalence will result in a high negative predictive value, or it may be because DVT that are missed by ultrasound have a relatively benign natural history.

## Conclusion

US has high sensitivity for proximal DVT, modest sensitivity for distal DVT and high specificity. Optimal sensitivity, particularly for distal DVT, is achieved by using duplex or triplex US, while optimal specificity is achieved by using compression US alone. US sensitivity appears to be higher in cohorts with higher DVT prevalence. However, these findings are subject to substantial unexplained heterogeneity and should be interpreted with caution. Evaluation of repeat US has been very limited and its' potential benefit is very uncertain.

## List of abbreviations

US = ultrasound

DVT = deep vein thrombosis

CI = confidence interval

## Competing interests

The authors are not aware of any conflicts of interest relating to this article. The United Kingdom Health Technology Assessment R&D Programme funded this project (reference number 02/03/01). The views and opinions expressed therein are those of the authors and do not necessarily reflect those of the UK Department of Health.

## Authors' contributions

SG designed the study, selected articles for inclusion, undertook meta-analysis, and wrote the paper. FS selected articles for inclusion, assisted with data extraction, and helped write the paper. ST and EvB helped to design the study, extracted data, assessed study quality, and helped write the paper. AS assisted with meta-analysis, undertook meta-regression, created the funnel plots, and helped write the paper. All the authors have seen and approved the final draft.

## Pre-publication history

The pre-publication history for this paper can be accessed here:



## Figures and Tables

**Table 1:** Results of meta-regression

**Variable**	**Sensitivity**	**Specificity**
Setting for recruitment	0.14	0.43
DVT prevalence	<0.001	0.11
Proportion of proximal DVT	0.04	0.14
Mean age	0.36	0.17
Proportion of males	0.66	0.24
Consecutive recruitment	0.78	0.55
Prospective study	0.26	0.24
Radiologist interpreted	0.064	0.52
Sonographer interpreted	0.52	0.44
Ultrasound performed blind to reference standard	0.44	0.52
Reference standard performed blind to ultrasound	0.59	0.56
Previous DVT excluded	0.92	0.039
Date of publication	0.057	0.84

p-value when included in random effect weighted meta-regression

**Table 2:** Pooled estimates of sensitivity and specificity stratified by US technique

	Sensitivity for all DVT	Sensitivity for proximal DVT	Sensitivity for distal DVT	Specificity
Compression only, N = 22	90.3% (88.4 to 92.0) P < 0.001	93.8% (92.0 to 95.3) P = 0.005	56.8% (49.0 to 66.4) P < 0.001	97.8% (97.0 to 98.4) P = 0.01
Colour Doppler only, N = 5	81.7% (77.4 to 85.5) P < 0.001	95.8% (85.7 to 99.5) P = 0.427	43.5% (23.2 to 66.5) P = 0.009	92.7% (89.7 to 95.1) P = 0.003
Continuous wave Doppler only, N = 16	81.1% (78.2 to 83.7) P < 0.001	87.8% (84.7 to 90.5) P < 0.001	41.8% (32.5 to 51.6) P = 0.015	84.0% (81.4 to 86.3) P < 0.001
Triplex, N = 25	91.1% (89.0 to 93.0) P < 0.001	96.4% (94.4 to 97.9) P < 0.001	75.2% (67.7 to 81.6) P < 0.001	94.3% (92.5 to 95.8) P < 0.001
Duplex, N = 28	92.1% (90.7 to 93.5) P < 0.001	96.5% (95.1 to 97.6) P < 0.001	71.2% (64.6 to 77.2) P < 0.001	94.0% (92.8 to 95.1) P < 0.001
Others, N = 4	93.3% (88.8 to 96.4) P = 0.338	-	-	96.0% (92.2 to 98.2) P < 0.001

95% CI in parentheses P-value = Chi-square test for heterogeneity

**Table 3:** Studies of repeat US

**Author & date of publication**	**Group**	**Number (%) of initial scans positive**	**Number (%) of repeat scans positive**	**Number (%) of positive scans (initial or repeat) confirmed by venography**
Heijboer, 1993 [109]	All patients	93/491 (19%)	7/397 (1.8%)	84/89 (94%)
Cogo, 1998 [110]	All patients	400/1702 (24%)	12/1252 (1.0%)	-
Sluzewski, 1991 [111]	All patients	67/174 (39%)	0/98 (0%)	-
Birdwell, 1998 [112]	All patients	63/405 (16%)	7/342 (2.0%)	23/28 (82%)
Birdwell, 2000 [113]	All patients	95/709 (13%)	9/521 (1.7%)	39/47 (83%)
**Studies of unselected patients combined**			**35/2610 (1.34%)**	**146/164 (89.0%)**
Bernardi, 1998 [116]	Positive D-dimer	260/946 (27%)	5/88 (5.7%)	-
Kraaijenhagen, 2002 [117]	Positive D-dimer	391/1739 (22%)	17/518 (3.0%)	-
**Studies of patients with a positive D-dimer combined**			**22/606 (3.63%)**	**-**
Wells, 1997 [114]	Intermediate Wells score	27/193 (14%)	3/166 (1.8%)	-
Tick, 2002 [115]	Intermediate or high Wells score & positive D-dimer	300/531 (57%)	13/83 (15.7%)	-
